# Primary Hepatic Paraganglioma Mimicking Hepatocellular Carcinoma: A Diagnostic Challenge and Literature Review

**DOI:** 10.7759/cureus.106531

**Published:** 2026-04-06

**Authors:** Michael K Konstantinidis, Dimitrios Vlachos, Dionysios Prevezanos, Ioannis Giannopoulos, Anastasios Stofas, Nikolaos Machairas, Georgios C Sotiropoulos

**Affiliations:** 1 Department of Liver Transplantation and Hepatobiliary Surgery, Laiko General Hospital of Athens, National and Kapodistrian University of Athens, Athens, GRC; 2 First Department of Pathology, National and Kapodistrian University of Athens, Athens, GRC

**Keywords:** hepatocellular carcinoma mimic, hypervascular liver tumor, liver resection, neuroendocrine tumor, primary hepatic paraganglioma

## Abstract

Primary hepatic paraganglioma (HPGL) is an exceptionally rare neuroendocrine tumor and represents a significant diagnostic challenge due to nonspecific clinical and radiological features. We report a rare case of primary HPGL initially misdiagnosed as a hypervascular liver tumor. A 72-year-old female was referred for evaluation of an incidentally detected hepatic mass. Preoperative assessment included contrast-enhanced computed tomography (CT), magnetic resonance imaging (MRI), and 18F-fluorodeoxyglucose positron emission tomography/CT (18F-FDG PET/CT). Surgical resection was performed, followed by histopathological and immunohistochemical examination. Postoperative functional imaging was used to exclude extrahepatic disease. Imaging revealed a 2.5-cm hypervascular lesion in segment IVb of the liver with moderate FDG uptake and no evidence of extrahepatic disease. The patient underwent open wedge resection, during which marked intraoperative blood pressure fluctuations were observed. Histopathology demonstrated a paraganglioma with characteristic organoid architecture and supportive immunohistochemical findings. Postoperative iodine-131 metaiodobenzylguanidine (MIBG) scintigraphy showed no additional lesions, confirming the diagnosis of primary HPGL. The postoperative course was uneventful, and no recurrence was detected at six-month follow-up. Primary HPGL is a rare entity that may mimic more common hypervascular hepatic tumors, leading to misdiagnosis. Surgical resection remains the treatment of choice. Intraoperative hemodynamic instability may serve as an important diagnostic clue, even in clinically nonfunctional cases. Long-term follow-up is recommended due to the uncertain malignant potential of these tumors.

## Introduction

Paragangliomas (PGLs) are rare neuroendocrine tumors that, along with pheochromocytomas (PCCs), form the spectrum of PCC-paraganglioma (PPGL) neoplasms [1]. These lesions originate from paraganglionic cell clusters derived from the neural crest and are broadly classified into two groups. The first includes head and neck paragangliomas (HNPGLs), which are associated with the parasympathetic nervous system. Unlike their sympathetic counterparts, HNPGLs are non-functional and typically act as chemoreceptors rather than secreting catecholamines [2]. The second category originates from sympathetic paraganglia, often in close proximity to sympathetic chains, and is capable of catecholamine secretion.

According to the World Health Organization (WHO), tumors arising from sympathetic tissue within the adrenal gland are termed PCCs, whereas those originating in extra-adrenal sympathetic tissue are classified as sympathetic paragangliomas (sPGLs) [3]. sPGLs typically develop along the longitudinal axis of the body, extending from the skull base to the pelvic floor, reflecting the distribution of sympathetic nerves [4]. Overall, PGLs encompass both HNPGLs and sPGLs, with reported frequencies of 55.2% in the retroperitoneum, 25.6% in the head and neck, 5.6% in the bladder, and 3.2% in the mediastinum [5,6]. In contrast, primary hepatic paragangliomas (HPGLs) are exceedingly rare, with 19 cases reported in the literature to date [7-25].

Clinical presentation varies depending on functionality. Commonly, HPGLs have no clinical manifestations and are found as incidental hepatic lesions during routine evaluation. Some patients manifest clinical features of catecholamine hypersecretion, such as hypertension, palpitations, and chronic headaches, whereas others present with symptoms related to mass effect on adjacent organs, such as abdominal pain and distention. Diagnostic confirmation is often difficult, as both clinical manifestations and radiological findings lack specificity [5,6].

In this report, we present the case of a 72-year-old female patient who was referred to our surgical department due to an incidentally diagnosed liver mass, initially misdiagnosed as hepatocellular carcinoma (HCC), but was found to be a primary HPGL.

## Case presentation

**Figure 1:**
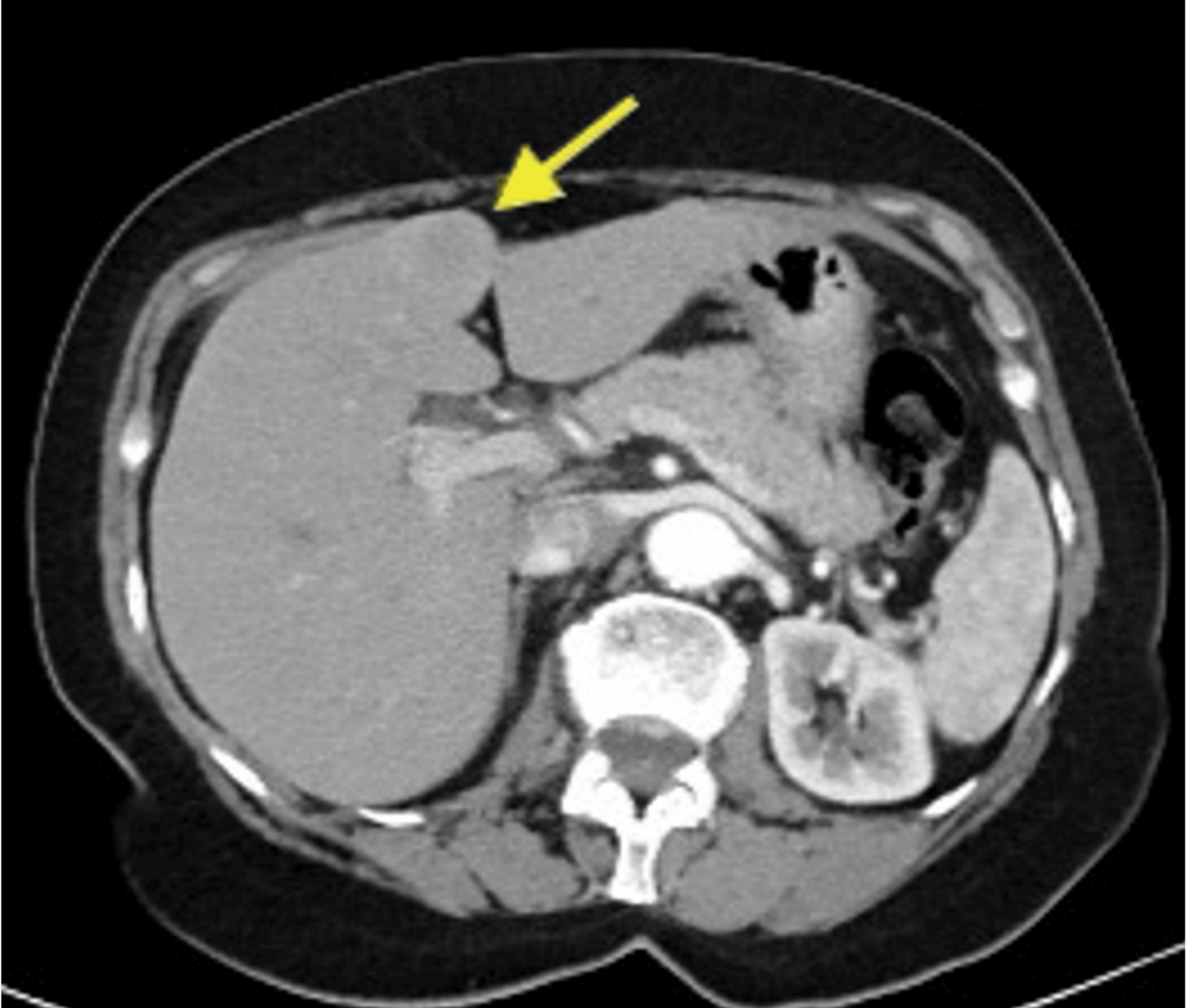
Contrast-enhanced computed tomography (CT), axial view, demonstrating a well-circumscribed 2.5-cm solid lesion in hepatic segment IVb (yellow arrow) with peripheral enhancement.

**Figure 2:**
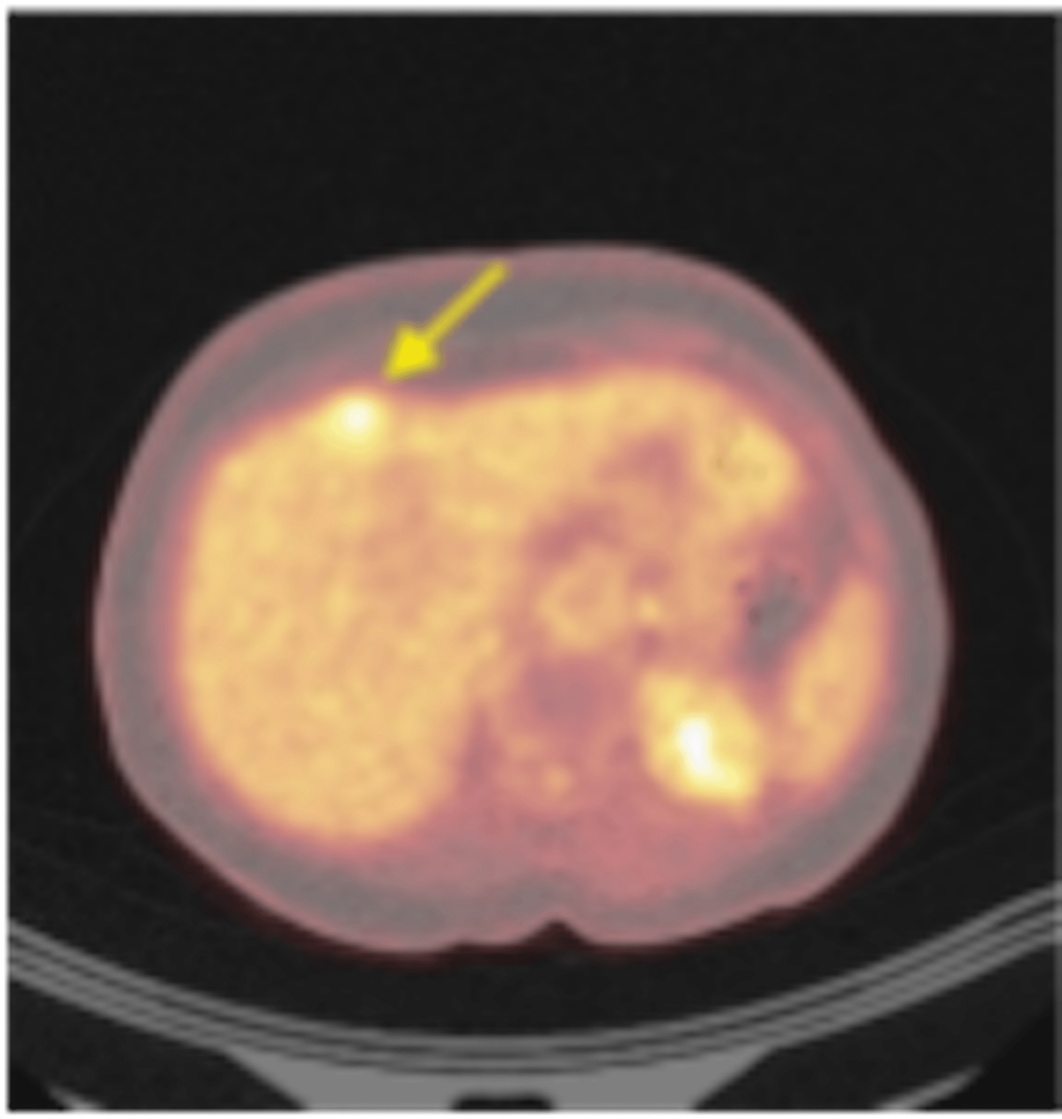
Axial fused 18F-fluorodeoxyglucose positron emission tomography/computed tomography (18F-FDG PET/CT) image showing focal moderate FDG uptake within the hepatic lesion (yellow arrow), without additional hypermetabolic foci.

**Figure 3:**
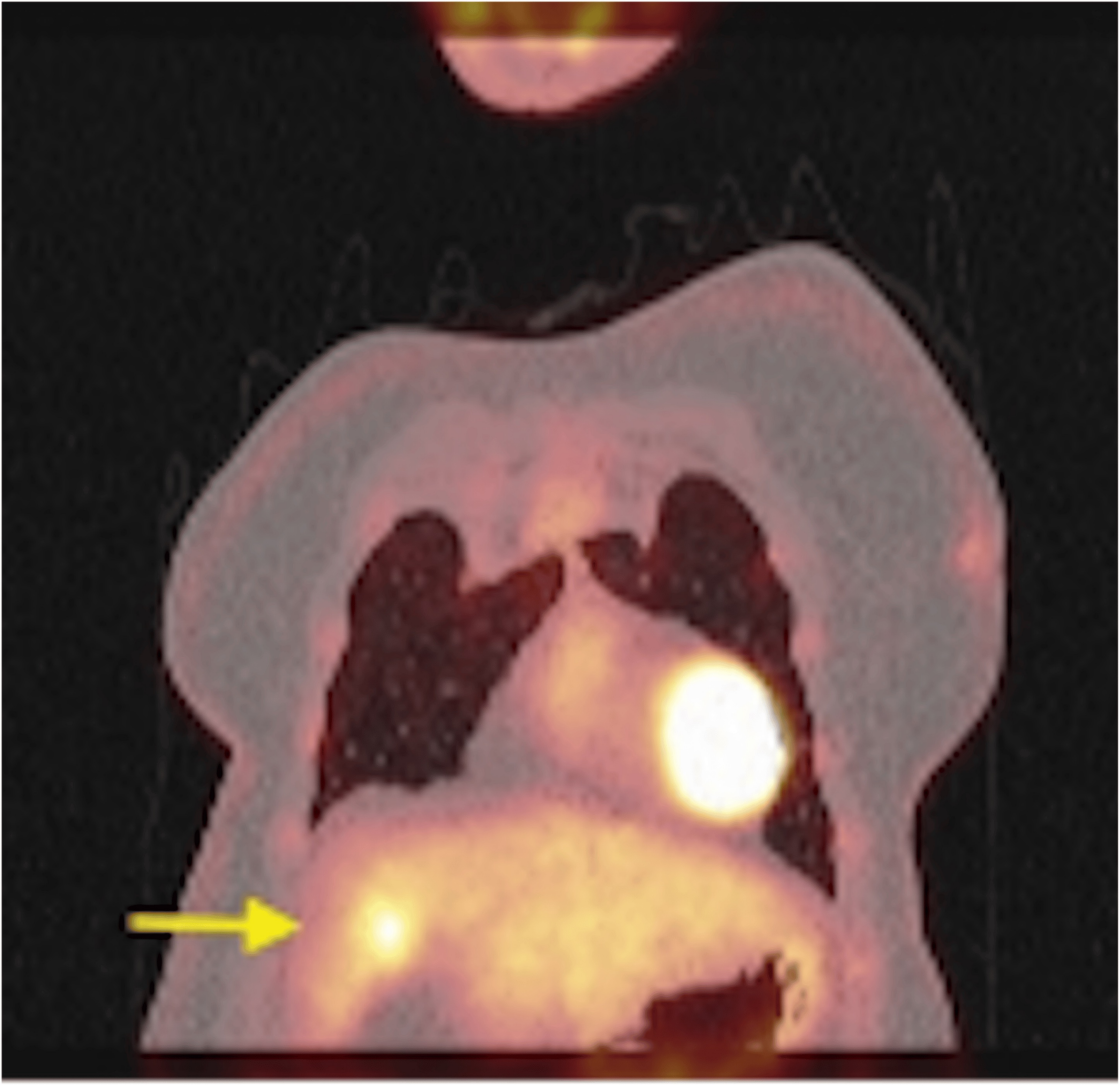
Coronal fused 18F-fluorodeoxyglucose positron emission tomography/computed tomography (18F-FDG PET/CT) image confirming isolated FDG uptake in the hepatic lesion (yellow arrow), with no evidence of extrahepatic disease.

A 72-year-old female patient with a history of hypertension, left internal carotid aneurysm, and seizures presented asymptomatically to our Hepatobiliary Unit following the incidental detection of a liver mass during routine evaluation. Initially, computed tomography (CT) scan and magnetic resonance imaging (MRI) revealed a 2.5 cm solid mass in segment IVb with peripheral enhancement (Figure 1). Given the indeterminate nature of the lesion and the need to exclude extrahepatic disease, positron emission tomography/CT (PET/CT) was subsequently performed, demonstrating moderate fluorodeoxyglucose (FDG) uptake without other localizations (Figures 2-3). Tumor markers and liver function were normal. The case was reviewed in a multidisciplinary tumor board, and surgical resection was recommended given the suspicion of a primary hepatic malignancy and the absence of extrahepatic disease.

She was treated successfully with open wedge resection of segment IVb, during which marked intraoperative blood pressure fluctuations were observed, particularly during tumor manipulation and resection. Postoperative course was uneventful, without complications, and she was discharged on the eighth day.

**Figure 4:**
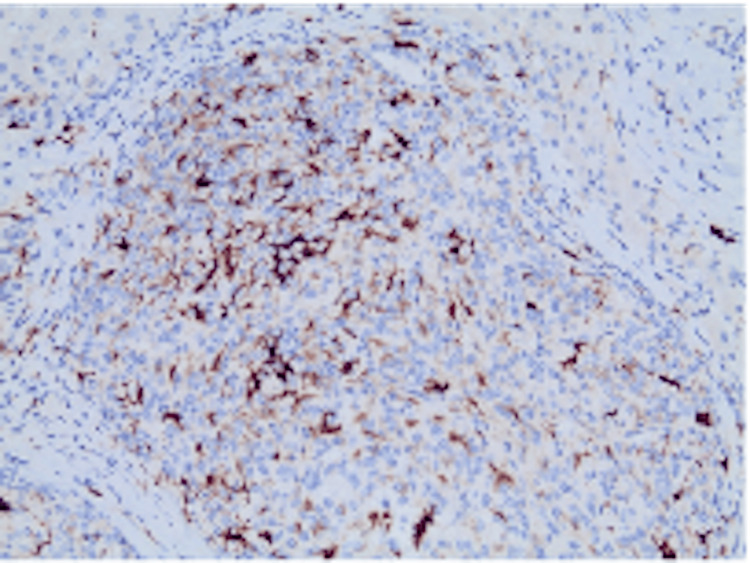
S100 staining in sustentacular cells and their cytoplasmic processes (IHC staining, 200× magnification). IHC: immunohistochemistry

**Figure 5:**
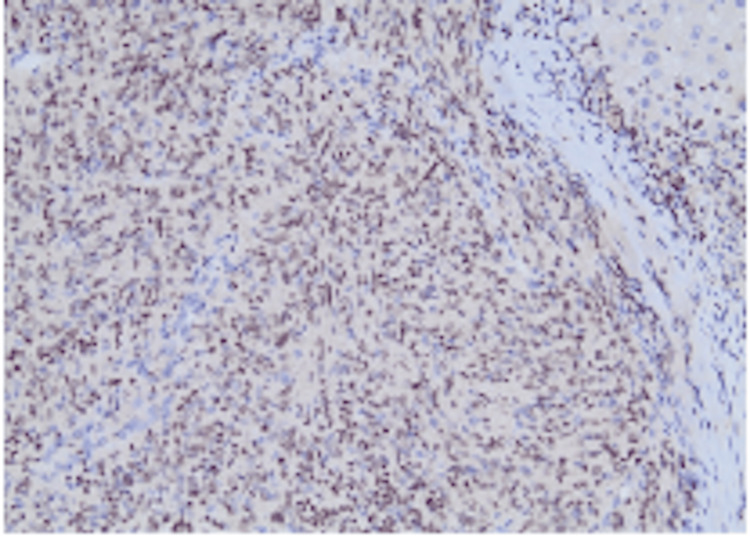
Paraganglioma chief cells with nuclear reactivity for GATA3 (IHC staining, 200× magnification). IHC: immunohistochemistry

**Figure 6:**
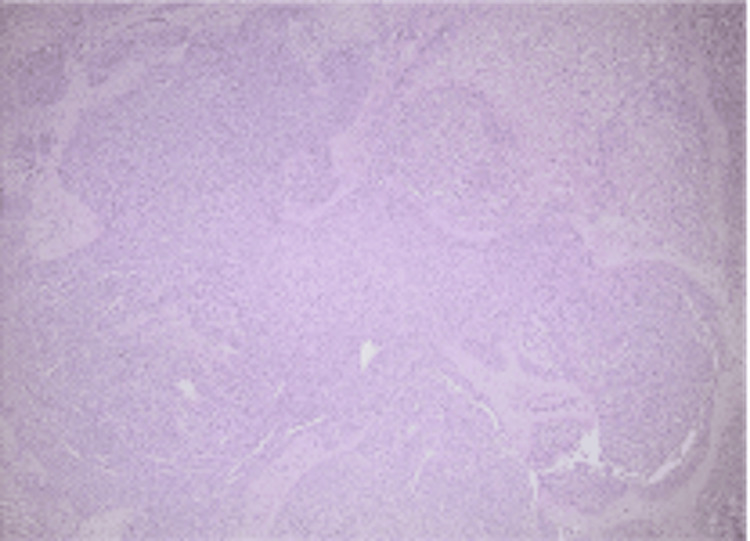
Paraganglioma with organoid-nested pattern (hematoxylin and eosin staining, 40× magnification).

**Figure 7:**
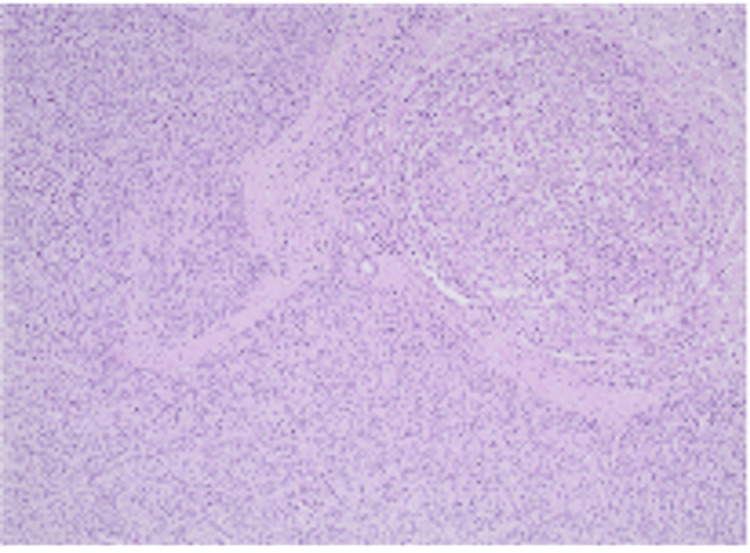
Paraganglioma with organoid-nested pattern (hematoxylin and eosin staining, 100× magnification).

Postoperatively, histopathological examination analysis as well as immunohistochemistry staining revealed a neoplasm with features compatible with PGL (synaptophysin+, CD57+, chromogranin-, Ki-67≈5%), with free resection margins (Figures 4-7). A subsequent iodine-131 metaiodobenzylguanidine (MIBG) scintigraphy revealed no additional lesions, supporting the diagnosis of primary HPGL. Six months postoperatively, she remains in excellent clinical condition with no signs of recurrence based on physical examination and radiologic work-up.

Owing to the exceptional rarity of hepatic involvement, this report adds to the scarce body of literature regarding its clinical presentation, operative management, and outcomes, while underscoring the necessity of including PGL in the differential diagnosis of hepatic neoplasms.

## Discussion

Primary HPGL is considered to be an exceptionally rare neuroendocrine tumor that arises from sympathetic paraganglionic tissue within the liver [1]. Even though it represents a common hepatic metastatic site from malignant PCCs and PGLs, it is rarely presented as a true primary hepatic involvement. To date, only a limited number of HPGL cases have been reported, underscoring the limited collective experience regarding its presentation, diagnostic approach, and optimal management. Collective experience remains sparse. Table 1 summarizes the available reported cases of primary HPGL and highlights several important clinicopathological characteristics relevant to the present case.

**Table 1:** Reported cases of surgically treated primary hepatic paraganglioma U/S: ultrasound; CT: computed tomography; MRI: magnetic resonance imaging; HCC: hepatocellular carcinoma; Ga^68^ FAPI PET/CT: gallium-68 fibroblast activation protein inhibitor positron emission tomography/computed tomography; N/A = not available

No.	Authors	Sex	Age	Clinical manifestations	Location	Size, cm	Primary diagnosis	Confirmed method	Follow-up (follow-up time)
1	Liao et al. (2018) [7]	Female	49	Asymptomatic	Segment VII, VIII	U/S: 2 at diagnosis, U/S: 5.7 x 4.9 after two years	HCC	Operation	No recurrence or metastasis in > two years
2	Li et al. (2022) [8]	Female	47	Hyper menorrhagia, Dizziness	Spiegelman lobe	MRI: 3.8 x 3.2 Intraoperative: 4 x 3	HCC	Operation	No recurrence or metastasis in one year
3	Lai and Zhong (2025) [9]	Male	22	Three days of intermittent right abdominal pain	Caudate lobe	MRI: 7.3 x 5.5 x 6.8	Hemangioma	Operation	N/A
4	Vella et al. (2022) [10]	Female	29	Asymptomatic	Along right hepatic vein and IVC	MRI: 6 at diagnosis, 12 after two years	HCC	Operation	No recurrence or metastasis in one year
5	Jiang et al. (2022) [11]	Female	34	Hypertension, palpitations, dizziness	Segment II, III	CT: 6.5 x 5.7. Specimen: 5 x 3.5 x 2.3	Paraganglioma	Operation	No recurrence or metastasis in one year
6	Kharroubi et al. (2022) [12]	Female	In her 30s	Chest discomfort, episodic perspiration, palpitations, dyspnea, fatigue, hypertensive episodes	Segment IV	MRI: 9 x 8 x 7	Paraganglioma	Needle biopsy	No recurrence or metastasis in four weeks
7	Lin and Hsu (2019) [13]	Female	41	Asymptomatic	Posterior segment of right lobe	Intraoperative: 3.5 x 4.5 x 4	Paraganglioma	Operation	No recurrence or metastasis in six months
8	You et al. (2015) [14]	Female	47	Hypertension	Segment III	CT: 3.6 x 3.4. Intraoperative: 4	HCC	Operation	Metastases below the right posterior lobe and in the spleen within 3.5 years of operation
9	Khan et al. (2011) [15]	Male	24	Six weeks of generalized bodyaches, headaches, fever, chills, sweating, abdominal pain, diarrhea	Right lobe	U/S: 15 x 18. CT: 20 x 18 x 14	Giant cavernous hemangioma or angiosarcoma	Needle biopsy	No recurrence or metastasis in three years
10	Reif et al. (1996) [16]	Female	42	Palpitation, headache, hypertension	Left lobe	CT: 4.5 x 3 Specimen: 4.5 x 5 x 3.8	Paraganglioma	Operation	No recurrence or metastasis in 14 months
11	Kim et al. (2018) [17]	Male	16	Right eye pain with blurriness, headaches, hypertensive crisis	Segment IV	MRI: 3.8 x 6.4 x 4.1	Paraganglioma	Operation (orthotopic liver transplantation)	No recurrence or metastasis in 2.7 years
12	Koh et al. (2013) [18]	Female	48	Six months of upper abdominal pain and distention	Segment V, VI	CT: 12 x 18 x 18	Cavernous hemangioma	Operation	N/A
13	Miller et al. (2021) [19]	Female	54	Three weeks of abdominal pain, bloating, early satiety; four weeks of fatigue, night sweats, weight loss	Caudate lobe	CT: 6.6	Paraganglioma	Operation	No recurrence or metastasis in long-term follow-up
14	Jaeck et al. (1995) [20]	Male	27	Sweating, hypertension	Segment VIII	MRI: 5	Pheochromocytoma	Operation	No recurrence or metastasis in one year
15	Chang et al. (2006) [21]	Male	37	Hypertension	Segment VI	CT: 6 x 5.5	HCC	Operation	No recurrence or metastasis in five years
16	Hong et al. (2013) [22]	Female	34	Asymptomatic	Segment VII	Specimen: 1.2 x 0.7	Metastatic from gastric tumor	Operation	No recurrence or metastasis in one year
17	Zhou et al. (2025) [23]	Female	55	Seven days of incomplete left eyelid closure, crooked right mouth corner	Left lobe, head and neck	Ga^68^ FAPI PET/CT: 1.4 x 1	Paraganglioma	Operation	N/A
18	Corti et al. (2002) [24]	Male	46	Hypertension	Segment VII, VIII	CT: 8	HCC	Operation	No recurrence or metastasis in nine years
19	Rimmelin et al. (1996) [25]	Male	24	Two years of hypertension and night sweats	Segment VIII	U/S: 5 x 3 CT: 5	Pheochromocytoma	Operation	No recurrence or metastasis in three years

Clinical manifestations of HPGL are highly variable but closely related to excess catecholamine secretion. Patients might exhibit symptoms such as palpitations, headaches, diaphoresis, and metabolic disturbances. However, as demonstrated both in the literature and in the present case, a substantial proportion of patients with HPGLs remain asymptomatic, with tumors discovered incidentally during imaging performed for unrelated reasons [2]. As demonstrated in the literature review, among the 19 presented cases of HPGL, four patients reported no clinical manifestations or blood pressure irregularities; three patients were incidentally found to have hypertension; eight patients reported clinical symptoms related to catecholamine excess such as headaches, dizziness, palpitations, night sweating and blurriness; two patients had upper abdominal pain; one patient also reported unilateral eye pain; and one also had unilateral incomplete eyelid closure. The patient presented herein was entirely asymptomatic at diagnosis, despite a known history of hypertension. This finding illustrates that functional activity may be clinically silent and undiagnosed preoperatively. Importantly, even in asymptomatic patients, dormant catecholamine secretion may become clinically evident due to tumor manipulation during surgery. In this case, this phenomenon was clearly observed during tumor handling and resection, where there was a marked fluctuation of intraoperative blood pressure. Similar hemodynamic instability has been described by Liao et al. [7] as well as by You et al. [14], making it a hallmark intraoperative clue to the diagnosis.

As for the radiological diagnosis of HPGL, it remains challenging due to the lack of specific pathognomonic imaging features. In the majority of reported cases, including the present one, HPGL was initially misdiagnosed as HCC or other benign hypervascular liver tumors, including cavernous hemangioma or adenoma. Characteristics of CT and MRI imaging mimick that of HCC, including hypoattenuating hepatic lesions and arterial phase hyperenhancement with portal or delayed phase washout [3]. Tumor size ranged widely, from small nodules less than 2 cm to giant masses exceeding 20 cm, with no consistent correlation between size and symptomatology. In the present case, MRI demonstrated a small hypervascular lesion with peripheral enhancement, while PET/CT showed moderate FDG uptake, findings that were nonspecific and favored a benign or malignant hepatic tumor rather than a neuroendocrine neoplasm.

One of the most reliable functional imaging modalities for PGLs is considered to be I-131 MIBG scintigraphy. Previous studies have reported high diagnostic accuracy in the detection of PCCs and PGLs, with sensitivities ranging from 77% to 95% and specificities varying between 95% and 100% [25]. In addition, somatostatin receptor-based imaging is also commonly used, given the neuroendocrine origin of these tumors that typically express somatostatin receptors on their cell surface. However, although radiolabeled octreotide can successfully localize lesions, its diagnostic accuracy is inferior to I-131 MIBG scintigraphy due to somatostatin receptor expression across a wide range of neuroendocrine neoplasms [26].

The diagnostic role of preoperative liver biopsy remains controversial. It carries potential risks, such as tumor rupture, bleeding, and catecholamine-induced hypertensive crisis. This procedure is often avoided because surgical resection is the preferred treatment for both HPGL and presumed HCC.

Definitive diagnosis is most commonly established postoperatively through histopathological and immunohistochemical examination. Histologically, HPGLs demonstrate characteristic features of PGL, including nested (“zellballen”) growth patterns composed of chief cells surrounded by sustentacular cells and a rich vascular network. Immunohistochemistry typically shows positivity for neuroendocrine markers such as synaptophysin and neuron-specific enolase, while sustentacular cells stain with S-100 [27].

In the present case, immunohistochemical staining was positive for synaptophysin and CD57, with a low Ki-67 index, consistent with a well-differentiated PGL. Chromogranin A expression may be variable, as observed in our case, and does not preclude the diagnosis. Importantly, confirmation of a primary hepatic origin requires exclusion of extrahepatic disease. Postoperative I-131 MIBG scintigraphy in our patient revealed no additional lesions, supporting the diagnosis of primary HPGL rather than metastatic disease.

Surgical resection remains the treatment of choice for HPGL and is generally associated with favorable outcomes when complete tumor removal is achieved. The surgical approach varies according to tumor size and anatomical location, ranging from limited wedge resections to major hepatectomies and, in rare cases, orthotopic liver transplantation [5]. In the present case, wedge resection was considered appropriate due to the lesion’s small size and favorable location. Notably, significant intraoperative blood pressure instability occurred during tumor handling, a finding reported in several previous reports [8,10,13] and suggestive of catecholamine release. This finding highlights the importance of meticulous anesthetic awareness, even in the absence of preoperative biochemical or clinical evidence of functional disease. It is necessary for doctors to be prepared for sudden hypertensive or hypotensive crises in order to prevent or mitigate complications, including hypertensive crisis and arrhythmia during surgery [28]. Finally, it is important for surgeons to remove the lesion carefully to reduce irritation to HPGLs [29].

Postoperative outcomes reported in the literature are generally favorable. Most patients, including the present case, experienced uneventful postoperative courses and remained disease-free during follow-up periods ranging from several months to many years. In only one case reported by You et al. [14], there was late metastatic disease several years after resection, suggesting that although HPGLs are typically indolent, malignant potential cannot be entirely excluded. Consequently, given the inability to reliably predict malignant behavior at diagnosis, careful postoperative monitoring with imaging and functional studies is essential. In the present case, the patient remained disease-free six months after surgery, consistent with the outcomes reported in most published cases.

## Conclusions

In summary, primary HPGL is an exceptionally rare and diagnostically challenging tumor with nonspecific clinical and radiological features. It may mimic more common hepatic neoplasms, frequently leading to misdiagnosis. Intraoperative hemodynamic fluctuations during tumor manipulation may serve as an important diagnostic clue, even in asymptomatic patients. Complete surgical resection appears to be an effective treatment option, although evidence remains limited due to the rarity of the condition. Given the inability to reliably predict malignant behavior, long-term surveillance should be considered. This case contributes to the limited existing literature and reinforces the importance of considering PGL in the differential diagnosis of hypervascular hepatic lesions, particularly when unexplained intraoperative hemodynamic instability is encountered.
